# A common framework for using and reporting consumer purchase data (CPD) in foodborne outbreak investigations in Europe

**DOI:** 10.1080/20008686.2021.2007828

**Published:** 2021-12-02

**Authors:** Solveig Jore, Uffe Christian Braae, Frederik Trier Møller, Ingrid Friesema, Karthik Paranthaman, Katri Jalava, Nathalie Jourdan-DaSilva, Emma Löf, Moa Rehn, Steen Ethelberg

**Affiliations:** a Department of Infection Control & Preparedness, Norwegian Institute of Public Health, Oslo, Norway; b Department of Infectious Disease Epidemiology and Prevention, Statens Serum Institut, Copenhagen, Denmark; c Centre for Infectious Disease Control, National Institute for Public Health and the Environment, Bilthoven The Netherlands; d Field Service, National Infection Service, Public Health England, Ashford UK; e Department of Mathematics and Statistics, University of Helsinki, Helsinki, Finland; f Départment des maladies infectieuses Santé Publique France (French National Public Health Agency), Saint-Maurice, France; g Communicable Disease Control and Health Protection, Public Health Agency of Sweden, Solna, Sweden; h Department of Public Health, Global Health Section, University of Copenhagen, Copenhagen, Denmark

**Keywords:** Consumer purchase data, foodborne outbreaks, epidemiology

## Abstract

Consumer purchase data (CPD) can be a powerful tool in the investigation of foodborne outbreaks through analyses of electronic records of food that individuals buy. The objective of this study was to develop a common framework for use of CPD in foodborne outbreak investigations using the expertise of European public health professionals from 11 European countries. We also aimed to describe barriers and limitations preventing CPD utilization.

CPD are mainly gathered from supermarket loyalty programmes, smaller consortia, and independent supermarkets. Privacy legislation governing CPD was perceived as the most crucial barrier for CPD usage, but still resolvable. The main practical challenges were obtaining consumer consent for CPD usage, the associated workload, data access, format, and analysis. Harmonising methods and reporting across countries, standardised consent forms and electronic consent methods were identified as solutions.

This guideline was developed to support outbreak investigators in overcoming barriers in using CPD, thereby increasing public health professionals’ application and value of this powerful investigation tool. In addition, we hope this framework will lead to more public health institutions, in collaboration with food safety authorities, making use of CPD in outbreak investigations in the future.

## Introduction

Consumer purchase data (CPD) are electronic records of consumer purchases, collected by the retailers (e.g. supermarkets) and stored in secure databases with key customer and product details [1]. CPD can be divided into two types; individual and population-level data. Individual-level data may be collected anonymously with unique purchases or as individual customer identifiable data. This data contains information on store location, purchase time, and product names. The population-level data are aggregated data, including store location, product type, and purchase time. For foodborne outbreak investigations, the individual-linked CPD is the primary focus. CPD can be subsequently linked to individuals by loyalty or membership cards, credit or debit cards, or by printed receipts or bank statements crosschecked with retailer databases.

**Figure 1.**
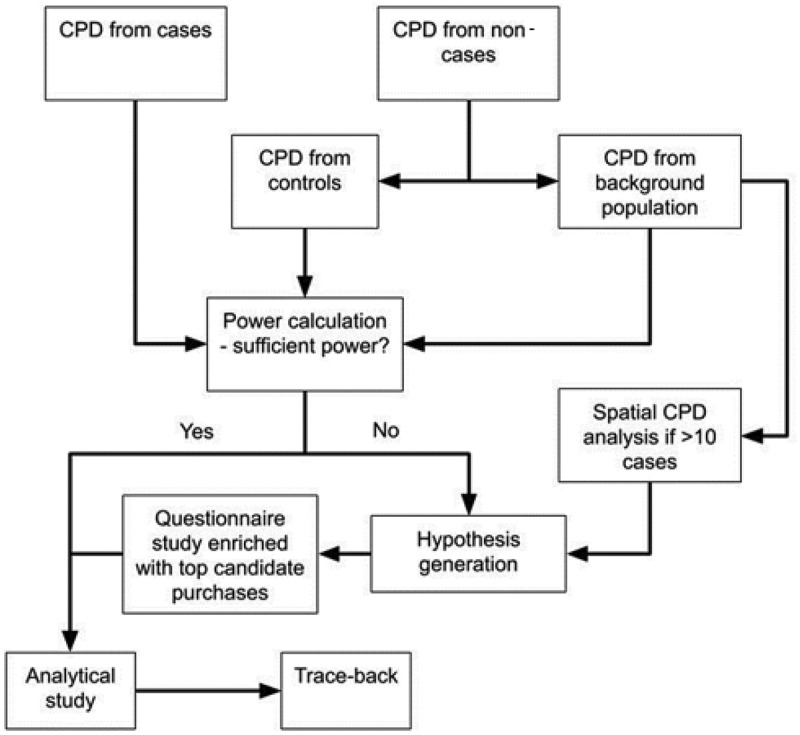
Pathways of foodborne outbreak investigations using consumer purchase data (CPD)

Foodborne diseases are a global, growing challenge with economic and public health impacts [2]. Early detection, implementation of control measures, notification, and investigation of foodborne outbreaks are key actions in identifying correct public health measures to reduce the burden of foodborne illness in the population. Outbreak investigations follow standardised steps. One of the critical challenges for analytical questionnaire data is the recall bias for the case patients and healthy referral participants to remember what they have purchased and eaten during the incubation period. Therefore, CPD are increasingly used as a tool in aiding foodborne outbreak investigations. However, there has been heterogeneity in the CPD usage methods among public health and food safety professionals. CPD is infrequently used in foodborne outbreak investigations [1], and if utilized, CPD methodology is often poorly described in the literature. Figure 1 shows pathways of foodborne outbreak investigations using consumer purchase data.

CPD is commonly used for hypothesis generation and gathers further evidence for any existing hypothesis [3–8]. CPD has also been used to conduct trace-back or perform analytical studies [9–14]. CPD has been shown to be a powerful tool in severe outbreaks with a single or few cases (e.g. botulism, listeriosis) when standard, statistical methods have failed to identify the causative vehicle [14,15]. Overall, heterogeneous ways to report on outcomes, description of CPD methodology and how CPD was collected, utilised or analysed have been major challenges [1]. Thus, the low use of CPD in foodborne investigations could be due to lack of awareness, knowledge and standardised protocols.

## Materials and methods

We conducted a questionnaire survey among public health and food safety professionals across Europe to elucidate barriers on the use of CPD in foodborne outbreak investigations under the EU-wide Horizon 2020 programme of the NOVA consortium (Novel approaches for design and evaluation of cost-effective surveillance across the food chain, https://onehealthejp.eu/jrp-nova/). Investigators from 11 European countries completed the survey, seven countries reported applying CPD in outbreak investigations, typically within national or regional public health institutes, or national food safety authorities. We also conducted focus group discussions among European disease outbreak public health specialists to further illuminate barriers and map existing usage of CPD in outbreak scenarios. These specialists were identified following a broad invitation to participate distributed via the Epiet Alumni Network (EAN) (https://www.ecdc.europa.eu/en/epiet-euphem/about/intro). The discussions were done as a series of three online meetings in 2019.

## Results

### General barriers in the use of CPD

**Figure 2.**
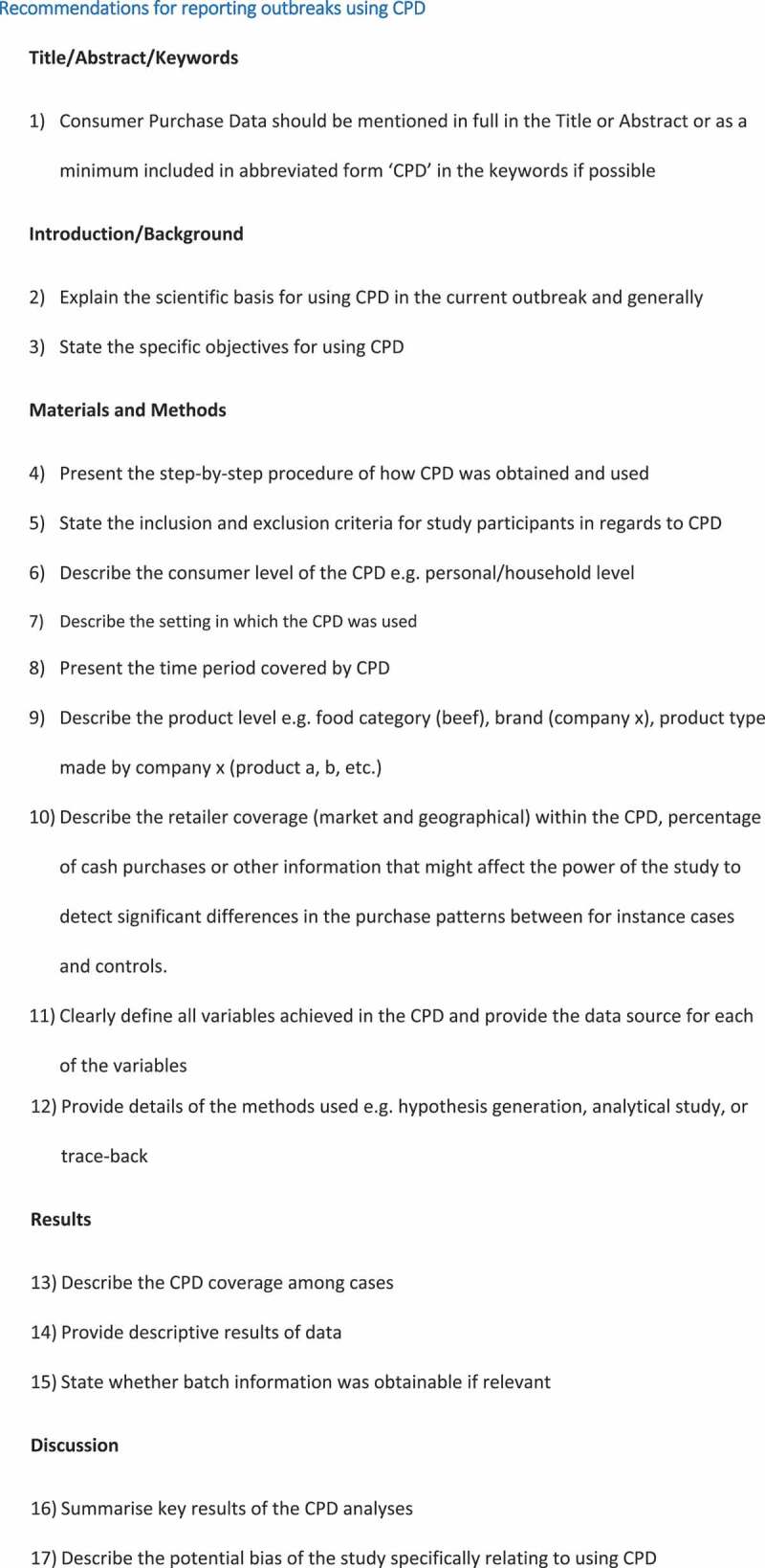
Standard operating procedures for reporting consumer purchase data (CPD) in regards to foodborne outbreak investigations

Several barriers preventing the use of CPD exist on an either regional, national or global scale. Barriers can be categorised as data availability and format constraints, feasibility constraints, or legislative constraints. Steps to be considered and reported if CPD were used in the outbreak investigation are presented in Figure 2.

### Data availability and format constraints

CPD are primarily gathered from supermarket loyalty programmes, mainly from larger supermarket chains, but smaller consortia and individual supermarkets may also hold valuable CPD. The availability of CPD differed by the size of the retailer, with smaller regional retailers less likely to store CPD, and more prone to unavailability issues. In contrast, large nationwide retailers have centralized servers, database storage facilities, and more employees with technical knowhow to assist the request. The format of the stored data varied from one retailer to another, complicating data handling and flexibility of obtaining data, especially if the data acquirement process is to be automated. Digitalized CPD from large, national supermarket chains will most likely become available for use in the near future. However, smaller retail chains or individual retailers might only have local or regional coverage and are therefore less likely to have CPD readily available.

### Feasibility constraints

Obtaining and analysing the data can be time consuming depending on several factors such as ease of data access, data format, and patient compliance, especially when CPD was in analogue format or used for hypothesis generation. Seven out of 11 European countries considered practical constraints or technical obstacles for obtaining and using CPD major issues, with workload being the primary obstacle. The number of steps and persons/institutions required before data access is granted is challenging when working with retailers. Subsequently, data handling and the use of different formats are significant obstacles in using CPD from different supermarkets. Optimising and automating the processes are considered priorities, as the workload on handling and processing data was perceived as important barriers to implementing CPD in outbreak investigations.

### Legislative constraints

The consensus was that insecurity regarding privacy legislation governing CPD is perceived as the most important barrier together with the ability to share consumer-level data with public authorities. Individual public health agencies need to work within the existing national legal position and public acceptability around data sharing. Obtaining consent from consumers can be complicated and time-consuming.

### Country specific differences

Some of the barriers encountered were partly country specific and determined the ease of using CPD. If a country had low coverage of supermarket chains and many small retailers, the challenge to gather CPD is much greater than in a country with fewer supermarket chains. The presence of a third-party collecting data from several supermarket chains in one database facilitates the use of CPD. Such a third party currently exists in Denmark, Sweden, Norway, and Finland. However, collection of grocery-related CPD was only widely used in Denmark. Within the EU, there were large differences in the proportion of cash-based transactions versus digital transactions, though loyalty card schemes often allow customers to register cash purchases as well.

We provide a practical guideline for using CPD in foodborne outbreak investigations; in summary, the steps include establishing data availability, ensuring informed consent from cases, acquiring data from retailers or third party providers, data formatting and background information issues and conducting the analysis.

### Guideline for using consumer purchase data (CPD) in outbreak investigations

This guide provides an overview of the required processes when using consumer purchase data (CPD) in foodborne outbreak investigations.

1. Establish data availability

Food purchases need to be paid by cards or payments identifiable by loyalty cards or equivalent to enable use of CPD in a foodborne outbreak investigation. The data needs to be stored on servers at the retailers or third-party providers. The data and data delivery mechanisms should be curated before an outbreak occurs to avoid discussions on what data to include, such as purchase date and time, quantity, and items of purchases.

(2) Secure informed consent

The requirements for informed consent vary between countries and retailers and should be legally sound and not delay the rapid use of CPD. Where required by national legal standards, informed consent on the usage of CPD must be obtained from cases and controls included in the outbreak investigation. In addition, the consent needs to cover the pathogens’ incubation time, disease reporting delay, shelf life of suspected products, and extended storage (e.g. freezing) before consumption.

(3) Acquire data from retailers or third-party provider

Data may be obtained directly from retailers or via a third-party provider given that the customers have obtained informed consent. Depending on data storage system, loyalty cards, and bank statements containing information on the purchases obtained from the consumers, it might be necessary to link consumers and their CPD. Third-party providers may collect CPD from selected supermarket chains based on bank/credit cards enabling linkage of CPD directly from a range of retailers simultaneously.

(4) Data formats and background information

The data may have various formats, from only product type to detailed information about each specific product purchased. Nevertheless, information on the proportion of retailers included in the investigation should be recorded. Depending on the data source, the level of product information will be recorded at three levels: level 1 – product type, level 2 – product name (commercial and generic), and level 3 – a unique barcode/item number/GTIN/EAN/UPC that identifies product ingredients, and storage information. The data available at card user/household level might require household-level consent if the household consists of more than one person performing the grocery shopping.

(5) Conduct analysis

Depending on data formats, percentages of cases consuming each product should be recorded. To find shared food items between cases hypothesis generation or analytical studies can be done. Based on CPD findings, the investigators should consider further investigations if needed. This might include food sampling investigations, analytical studies or trace-back and trace-forward studies. Conducting especially microbiological analysis on suspected food items is of vital importance.

## Discussion

### Standardised reporting for outbreaks using CPD

Past studies have reported CPD usage in very different ways [1]. This clearly illustrates the need for a standardized framework for investigating and reporting. Our study presents a direct and practical approach in applying and reporting CPD under various outbreak settings. Figure 2 illustrates the steps that should be considered and reported if CPD is used in the outbreak investigation. First, it is important to report the market coverage (how large a proportion and geographical coverage of the monetary market the retailers providing CPD cover), unless other data indicates a specific supermarket chain as the source of the outbreak. In addition, retailer coverage might be important in assessing the geographical spread of cases and the strength of an association. Also, the variables typically available in the CPD such as product type, product name, batch number are crucial to understand the strength and limitations of the investigation. In a previous review [1], we could identify only one study [9], which thoroughly described the CPD variables.

We would encourage national authorities to arrange dedicated workshops with retailers and public health/food safety agencies to agree and establish general procedures needed for outbreak investigations. Ideally, such agreed procedures should be published in relevant outbreak response documents/plans in the public domain to ensure transparency and public access. Our framework can assist wide implementation and ease access to CPD usage while reporting guidelines provide consistency in publications. However, despite a structured framework (Figure 1), practical obstacles like country-specific barriers need to be addressed. Nonetheless, we perceive this framework as a necessary and significant step forward. Purchases are increasingly cash free and the possibilities of applying CPD in foodborne outbreak investigations and wider consumer health studies, are thus increasing. CPD data has proven powerful when used, even with limited access to CPD [1]. As CPD will cover a larger percentage of the population and from multiple retailers, CPD will likely gain importance as a starting tool after hypothesis generation in foodborne outbreak investigations, rather than as a tool used when all other sources seem exhausted. As the CPD becomes readily available, foodborne outbreak investigation teams should explore the possibilities of this type of data more frequently.

### Strength and limitations of CPD used in outbreak investigations

Usage of CPD is also beneficial for retailers as it allows for rapid identification of implicated products and robust, targeted control measures. CPD can also assist in excluding incorrectly hypothesized products. CPD are potentially very powerful in overcoming recall bias regarding foods or product type, especially when multiple types of the same food are suspected.

Naturally, there are, also inherent limitations when using CPD. Regardless of the CPD quality, the purchase of a product does not necessarily equal consumption. More importantly, lack of purchase does not necessarily mean lack of consumption; CPD tend to primarily capture foods consumed in the household. In addition, defining the CPD exposure period can also be a challenge, as purchase date does not necessarily equal consumption date. Public health and food control professionals need to consider the exposure period of interest, based on disease epidemiology, hypothesized food product, and shopping patterns. Another challenge is the sometimes long shelf life of products and the potential to freeze certain products. Finally, it cannot be ruled out that the consumers have bought products at retailers not providing purchases or not using their loyalty card every time, variably paid with cash only, or conducted otherwise non-registered purchases.

CPD is a relatively new methodology, but considerable differences between countries exist in the application of CPD. Current practice has evolved in different countries based on legal and regulatory policies, relationships between public health/food safety agencies and retailers, and capacity to undertake such investigations. This framework for use of CPD is based on a broad consensus among experienced outbreak investigators and were critically reviewed by experienced public health and food safety professionals. Outbreaks necessitate rapid access and analysis to inform control measures. Public health authorities and food safety authorities should work with their retailers and other data holders to consider how CPD could be used rapidly in outbreak situations within the legal framework. Systematically developed guidelines can improve accessibility and enhance use of CPD. Further, it can also aid in improving the transparency and comparability of studies and thus impact the quality of research done in this domain.
